# Mid-term health-related quality of life in community-acquired bacterial meningitis survivors; the COMBAT study

**DOI:** 10.1371/journal.pone.0281544

**Published:** 2023-03-23

**Authors:** Claire Della Vecchia, Josée Vicentia Ebah, Sarah Tubiana, Thomas Guimard, Lionel Piroth, Sylvain Jaffuel, Isabelle Gorenne, Bruno Mourvillier, Bruno Hoen, Xavier Duval, Marie Préau

**Affiliations:** 1 INSERM, Unité U1296 « Radiations: Défense, Santé, Environnement », Lyon, France; 2 Sorbonne Université, INSERM, Institut Pierre Louis d’Épidémiologie et de Santé Publique, Nemesis team, Paris, France; 3 IAME, INSERM, Paris University, Paris, France; 4 Inserm Clinical Investigation Centre 1425, Paris, France; 5 Infectious Diseases and Emergency Department, Centre Hospitalier de La Roche sur Yon, La Roche-sur-Yon, France; 6 INSERM CIC 1432, Infectious Diseases Department, CHU, University of Burgundy, Dijon, France; 7 Department of Infectious Diseases, Brest University Hospital, Brest, France; 8 AP-HP, Department of Epidemiology, Biostatistic and Clinical Research, Inserm CIC-EC 1425, Bichat Hospital, Paris, France; 9 Medical and Infectious Diseases Intensive Care Unit, AP-HP, Bichat Hospital, Paris University, Paris, France; 10 CHU de Nancy, Hôpitaux de Brabois, Service de Maladies Infectieuses et Tropicales, Vandœuvre-lès-Nancy cedex, France; 11 INSERM, F-CRIN, Innovative Clinical Research Network in Vaccinology (I-REIVAC), Paris, France; MRC/UVRI & LSHTM Uganda Research Unit, UGANDA

## Abstract

**Background:**

Community Acute Bacterial Meningitis (CABM) is a rare infectious disease leading to important impairments. Our aim was to describe CABM survivors’ quality of life (QOL) 12 months post-CABM and to assess its associations with CABM sequelae.

**Methods:**

Patients included in the CABM COMBAT cohort were evaluated one year after the CABM episode. Data were collected by questionnaire, via phone calls with the patients. The WHOQOL-BREF was used to measure CABM survivors’ QOL. Hierarchical multivariate linear regressions were performed.

**Results:**

Study population was composed of 284 patients. At 12 months, 53.9% (153/284) reported at least incident headache/worsening headache intensity at 12 months post-CABM, and/or incident hearing impairment, and/or unfavourable disability outcome (GOS). Unfavourable disability outcome was associated with lower physical health QOL (B = -30.35, p<0.001), lower mental health QOL (B = -15.31, p<0.001), lower environmental QOL (B = -11.08, p<0.001) and lower social relationships QOL (B = -9.62, p<0.001). Incident headache/worsening headache since meningitis onset was associated with lower psychological health (B = -5.62, p = 0.010). Incident hearing impairment was associated with lower physical QOL (B = -5.34, p = 0.030). Hierarchical regressions showed that CABM impairments significantly increase explanatory power of multivariate models (for physical health R^2^ change = 0.42, p<0.001, for psychological health R^2^ change = 0.23, p<0.001, for social relationships R^2^ change = 0.06, p<0.001 and for environment domain R^2^ change was 0.15, p<0.001).

**Conclusions:**

12 month-CABM burden is heavy. Early detection and management of CABM impairments should be performed in clinical practice as early as possible to optimize patients’ psychological and psychosocial functioning.

**ClinicalTrial. Gov identification number:**

NCT01730690.

## Background

Community Acute Bacterial Meningitis (CABM) in adults is a rare infectious disease. Its annual incidence is between 4 and 6 cases per 100,000 adults in high-income countries [1, 2]. Despite the advances in CABM care and treatment [3], this is a devastating disease, with a mortality rate of about 20% [4], and it causes important sequelae (focal neurological deficits, cognitive impairment, hearing loss [5, 6], headache [7]) in about one-third of the survivors [8]. Those sequelae may have an impact on patients’ lives and notably on patients’ psychological and psychosocial functioning.

The World Health Organization (WHO) defines quality of life (QOL) as "an individual’s perception, of his/her place in the existence, in the context of the culture and value system in which she/he lives, in relation to his/her objectives, expectations, standards and concerns" [9]. Measuring QOL, and its associated factors, is important as it helps understanding the impact of health status on the different aspects of a patient’s daily life. QOL is a complex concept, that not only refers to health but also refers to interactions people have with their living environment [10].

In the specific case of CABM in adults, few studies have specifically investigated subjective QOL, most of them focused on acute clinical management and prognostic factors [11–14] or on clinical outcome (degree of recovery) such as Glasgow Outcome Score (GOS) [15]. The few studies investigating QOL in adults after CABM showed that patients’ QOL was lower than controls [16] especially for physical and social functioning [17]. A previous study based on our study population (COMBAT study), showed that 12 months after meningitis onset, more than one in two CABM patients had physical Health-Related Quality of Life (HRQOL) < 25^th^ percentile of the score in general French population [7]. These findings led us to specifically investigate the issue of quality of life, and its determinants in CABM adult survivors, 12 months after meningitis. We assume that patients’ background factors (sociodemographic factors, medical status), indicators related to CABM (clinical course: seizures, coma, admission in ICU, microorganisms involved) and CABM impairments (level of people’s recovery, hearing problems and headache) have a significant impact on people’s psychosocial and psychological functioning. A better understanding of factors influencing QOL after CABM could help identify some ways to improve CABM survivors’ support and then patients’ health status by considering their personal experience of CABM.

## Methods

### Study design and subjects

COMBAT study is a national prospective multicenter cohort study (comprising 69 hospitals) that enrolled adults (age ≥18 years) with CABM between February 2013 and July 2015 [7]. Patients or their legal representatives received written information about the study. Only those who gave consent were definitely enrolled. Non-French speaking patients, those lost to follow-up at 12 months, and patients opposed to study participation were excluded from the sample [7].

### Data collection

Patients’ follow-up lasted one year after CABM. At 12 months, a telephone call was made by a trained researcher to ask patients about their health status and subjective HRQOL. When it was not possible to interview the patients directly, interviews were addressed to 1)patient’s proxies and 2)general practitioner. In case of non-response, the phone call was repeated 3 times in a period of 3 weeks; in case of failure after 3 phone call attempts, vital status was obtained using the French Epidemiology Centre on Medical Causes of Death (CepiDc) database.

#### Data collected at hospitalization time

During the initial meningitis hospitalization, background characteristics, health status and clinical course were recorded electronically in the case report form. The presence of a pre-existing hearing impairment and headache were also collected at the time of hospitalization.

#### Data collected one year after CABM

At one year after CABM, disability outcome was assessed according to the GOS in its French version [18]. We considered GOS scores related to vegetative state, severe disability and moderate disability (scores from 2 to 4 in the initial scale) as unfavourable disability outcomes and a score of 5, corresponding to a good recovery, as a favourable outcome [1]. Patients were also asked about eventual incident hearing disorders at 12 months from meningitis. Thus, hearing impairment has been grouped into 3 categories: 1)no hearing impairment, 2)hearing impairment prior to meningitis, 3)onset of hearing impairment since meningitis. A reassessment of eventual headaches at 12 months post-CABM was also performed to detect onset of headaches since meningitis. Thus, headache were grouped into 2 categories: 1)no headache, disappearance of headaches at 12 months, constant or decreasing intensity from before meningitis to 12 months, 2)onset of headaches at 12 months or worsening intensity at 12 months.

Outcome variables for this study were patients’ perception of the four QOL’s domains. Data were collected 12 months after meningitis onset using the WHOQOL-BREF questionnaire (24 items), an abbreviated version of the WHOQOL-100 developed by the WHOQOL-group [19]. This questionnaire contains two items related to overall QOL and satisfaction with health, and the other items exploring four QOL’s domains: physical QOL (pain and discomfort, energy and fatigue, sleep and rest, mobility, treatment dependence, daily life activities); psychological QOL (positive and negative feelings, self-esteem, body image, satisfaction with cognitive capacities, spirituality/religion); social QOL (interpersonal relationships, social support, sexual activity); environmental QOL (satisfaction with physical security, home, pollution, noise, information access needed in daily life, accessibility to and quality of health care, spare-time activities). The two items related to overall QOL and general health were rated on a Likert scale from 1: very poor to 5: very good and 1: very dissatisfied to 5: very satisfied respectively. For the four QOL’s domains, scores range from 0 to 100, higher values indicating better QOL. The WHOQOL-BREF was translated and validated in French and showed good psychometrics properties [20].

### Statistical analysis

Baseline and outcome characteristics were described. Categorical variables were summarized as counts (percentages). Continuous variables were expressed as medians with interquartile ranges (IQR), mean with standard deviation and minimum and maximum to describe QOL’s domains scores. We checked the internal consistency of each domain of the WHOQOL-BREF by calculating Cronbach’s alpha.

We investigated factors associated with QOL among the following variables: patient’s background characteristics, initial clinical presentation, CABM impairments at 12 months from meningitis onset: disability scores (GOS), hearing impairment and headache. In a first step, bivariate analyses were performed between our outcome variables and independent variables using parametric (ANOVA, Student’s t-test and Pearson’s correlation coefficient) or non-parametric equivalent tests if appropriate. We selected variables associated with outcome variables at a threshold of 20% (p<0.20) for multivariate analysis, we also included in final regression analysis potential confounding variables (mainly sociodemographic variables). To analyze the relationship of sociodemographic factors, medical data (medical background and CABM clinical course) and CABM impairments at 12 months (GOS, headache, hearing impairment) on the four WHOQOL-Bref domains, hierarchical multivariate linear regression analyses were performed. We chose to use the linear score, but we could have used a threshold as used in some works. However, to our knowledge, there is no validated threshold for identifying people with impaired QOL when using the WHOQOL scale. For each independent variable of the model, unstandardized coefficients with their associated p-value were presented. Determination coefficients (R²) showing the explained difference in variance of QOL’s domains explained by each group of associated variables was presented, as well as statistical significance of the R^2^ improvement with an analysis of variance (ANOVA). Linear regression assumptions were checked, as well as multicolinearity issues. The level of significance for the multivariate model and ANOVA was set at the 5% p-value threshold.

Analyses were performed by using RStudio 1.2.5033 software [21].

#### Ethics and regulatory issues

This study was registered at clinicaltrials.gov (identifier: NCT01730690) and received ethics approval by the “Comité de Protection des Personnes Ile de France CPP 4” (IRB 00003835) (2012-16NI), and the “Commission Nationale de l’Informatique et des Libertés” (CNIL) (EGY/FLR/AR128794).

## Results

### Baseline and outcome characteristics

**Fig 1:**
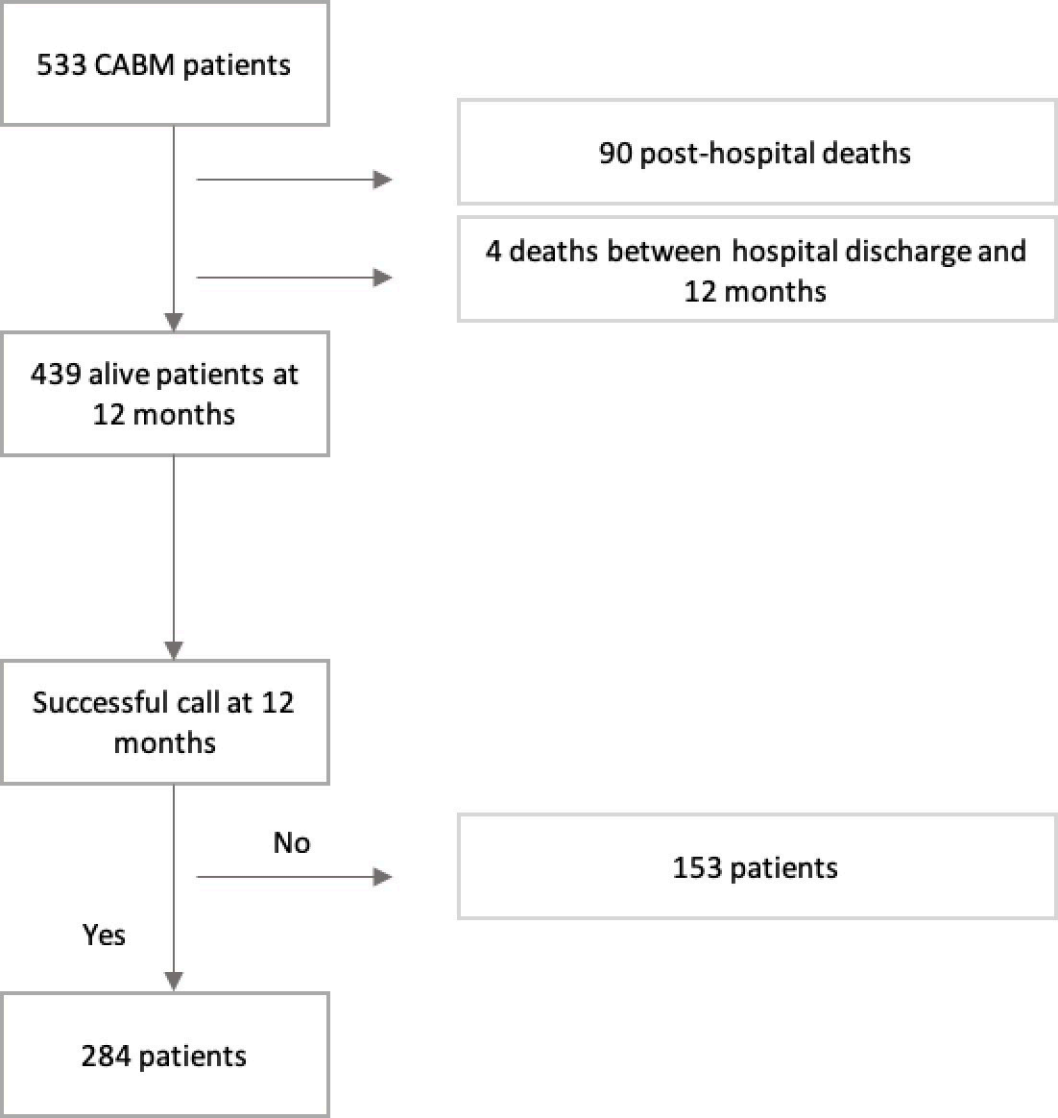
Flow chart of the study sample (N = 284), COMBAT France, 2013–2015.

A total of 533 CABM patients were included in the study, 94 did not survive at 12 months. Among the 439 survivors, 284 patients (i.e. our study population) answered the questionnaire at 12-month follow-up (Fig 1); 89.5% (248/277) of patients answered the questionnaire by themselves, for 8.3% (23/277) of patients, data were obtained through proxies or general practitioners, and for 2.2% (6/277), patients were helped by a proxy to answer. Male gender (p = 0.048), excessive alcoholic consumption (p = 0.028), and unemployed people (p = 0.044) at inclusion were significantly less likely to be represented in the 284 patients who were successfully contacted for this study (data not shown).

Among the 284 successfully contacted patients, 52.1% (148/284) were men, median age was 56.2 years (IQR: [38.1–66.0]), 43.8% (124/283) were in employment, 39.9% (113/283) were retired, 8.1% (23/283) were students and 8.1% (23/283) were unemployed. Regarding participants’ health status, 37.4% (104/278) had a chronic disease, 11.4% (32/280) have or had a cancer, 6.5% (18/279) had recurrent meningitis, 24.2% (67/277) were active smokers, 12.5% (35/281) were alcoholics and 4.6% (13/280) suffered from undernutrition. Concerning CABM clinical course, in 48% (132/275) of patients causative microorganism was Streptococcus pneumoniae, the large majority of patients received corticotherapy (72.7%, 205/282) and were admitted in ICU (76.2%, 215/282). Moreover, 15.5% (43/278) had seizures and 14.2% (40/282) were in coma (Table 1).

**Table 1:** Baseline characteristics at admission, CABM 12-month impairments, COMBAT France, 2013–2015.

Variables		n/N (%) or Median (IQR)
Background characteristics		
Age at meningitis		56.2 (38.1–66.0)
Male sex		148/284 (52.1)
Social professional categories		
	Executives^1^	36/283 (12.7)
	Employees^2^	88/283 (31.1)
	Students	23/283 (8.1)
	Retired	113/283 (39.9)
	Unemployed	23/283 (8.1)
**Health status**		
Chronic diseases		104/278 (37.4)
Cancer (<5 years)		32/280 (11.4)
Alcohol abuse		35/281 (12.5)
Active smoking		67/277 (24.2)
Recurrent meningitis		18/279 (6.5)
Undernutrition		13/280 (4.6)
**Clinical course**		
Causative microorganisms		
	*Streptococcus pneumoniae*	132/275 (48)
	*Neisseria meningitidis*	71/275 (25.8)
	Others	72/275 (26.2)
Corticoids		205/282 (72.7)
Admission in ICU		215 /282 (76.2)
Seizures		43/278 (15.5)
Coma		40/282 (14.2)
**12-month follow-up CABM impairments**		
GOS		
	Favourable outcome	191/277 (68.9)
	Unfavourable outcome	86/277 (31.0)
Headache		
	No headache, disappearance, decrease at 12 months	211/269 (78.4)
	Incident headache, worsening intensity at 12 months	58/269 (21.6)
Hearing impairment		
	No hearing impairment before and since meningitis	139/279 (49.8)
	Hearing impairment prior to meningitis	62/279 (22.2)
	Onset of hearing impairment since meningitis	78/279 (28.0)

^1^ executive, senior executive, teacher, liberal profession;

^2^ employee, worker

At 12-month follow-up, 31% (86/277) of participants had an unfavourable disability outcome (GOS score <5), 21.6% (58/269) reported incident headache or worsening headache intensity and 28% (78/279) declared incident hearing impairment (Table 1). Overall, 53.9% (153/284) of patients presented at least one of these 3 disorders.

Regarding outcome variables, median scores of QOL were 71.4 (Q1-Q3: 53.6–85.7) for physical health, 66.7 (Q1-Q3: 58.3–75.0) for mental health, 75.0 (Q1-Q3: 65.6–84.4) for environmental QOL, and 75.0 (Q1-Q3: 66.7–83.3) for social relationships, with higher values indicating better QOL in the domain (range 0–100) (Table 1). Median score for overall QOL was 4 (Q1-Q3: 3–4) and 4 (Q1-Q3: 2.25–4) for general health (Table 2). Internal consistency of QOL’s domains are good for physical health and environmental domains (α = 0.840 and 0.750 respectively) and acceptable for psychological and social relationships domains (α = 0.614 and 0.630 respectively).

**Table 2:** CABM participants’ WHOQOL-Bref scores at 12 months from CABM.

WHOQOL-Bref domains	Median (IQR)	Mean (SD)	Min-Max
Overall QOL	4 (3–4)	3.8 (0.8)	1–5
General health	4 (2.25–4)	3.4 (1.1)	1–5
Physical Health	71.4 (53.6–85.7)	68.0 (21.3)	7.1–100
Mental Health	66.7 (58.3–75.0)	65.4 (15.6)	12.5–100
Environment	75.0 (65.6–84.4)	74.1 (14.1)	0–100
Social Relationships	75.0 (66.7–83.3)	73.2 (17.1)	0–100

### Determinants of impaired QOL at 12 months

Table 3 shows the determinants of patients’ QOL at 12 months from CABM. Variables included in the models were those associated with QOL’s domains at a 20% p-value threshold (p<0.20) (S1 Table) and confounding variables. Hierarchical linear regression analyses revealed that CABM impairments were the most important predictors of the four domains of QOL at 12 months (Table 3).

**Table 3:** Factors associated with QOL in adults with CABM (N = 284), COMBAT France, 2013–2015.

Variables		Physical health QOL Multivariate analysis (B, p-value)	Psychological health QOL Multivariate analysis (B, p-value)	Social relationships QOL Multivariate analysis (B, p-value)	Environmental QOL ^1^ Multivariate analysis (B, p-value)
**Group 1 (sociodemographic factors)**		**R^2^ = 0.10**	**R^2^ = 0.04**	**R^2^ = 0.09**	**R^2^ = 0.07**
Sex (ref. Male)		-4.23 (0.044)*	-4.36 (0.015)*	-1.64 (0.443)	-1.94 (0.276)
Age		0.01 (0.969)	0.12 (0.153)	-0.03 (0.736)	0.08 (0.323)
Social professional categories					
	Executives ^2^	Ref.	Ref.	Ref.	Ref.
	Employees ^3^	0.79 (0.816)	-0.51 (0.866)	-0.86 (0.806)	-3.17 (0.246)
	Students	4.00 (0.432)	0.89 (0.843)	1.15 (0.822)	-0.40 (0.926)
	Retired	-6.36 (0.095)	-3.63 (0.293)	-3.01 (0.447)	-3.13 (0.363)
	Unemployed	-3.08 (0.517)	-1.08 (0.793)	-12.60 (0.008)*	-9.27 (0.054)
**Group 2 (health status, CABM clinical course)**		**R^2^ = 0.13**	**R^2^ = 0.06**	**R^2^ = 0.13**	**R^2^ = 0.08**
Chronic diseases / Cancer (<5 years) (ref. No)		-4.82 (0.029)*	/	-0.79 (0.728)	/
Alcohol abuse (ref. No)		-3.40 (0.338)	/	-4.17 (0.212)	-2.98 (0.354)
Active smoking (ref. No)		-3.16 (0.211)	/	/	-0.41 (0.838)
Causative microorganisms					
	*Streptococcus pneumoniae*	*Ref*.	*/*	*/*	*/*
	*Neisseria meningitidis*	-1.31 (0.650)	/	/	/
	Others	2.10 (0.395)	/	/	/
Admission in ICU (ref. No)		/	/	-2.35 (0.315)	-3.28 (0.087)
Seizures (ref. No)		/	-2.41 (0.338)	-5.94 (0.046)*	/
Coma (ref. No)		/	-4.39 (0.089)	/	/
**Group 3 (12-month follow-up CABM impairments)**		**R^2^ = 0.55**	**R^2^ = 0.29**	**R^2^ = 0.20**	**R^2^ = 0.25**
GOS					
	Favourable outcome	Ref.	Ref.	Ref.	Ref.
	Unfavourable outcome	-30.35 (<0.001)**	-15.31 (<0.001)**	-9.62 (<0.001)**	-11.08 (<0.001)**
Headache					
	No headache, disappearance, decrease at 12 months	Ref.	Ref.	Ref.	Ref.
	Incident headache, worsening at 12 months	-4.51 (0.069)	-5.62 (0.010)*	-1.58 (0.524)	-2.05 (0.298)
Hearing impairment					
	No hearing impairment	Ref.	Ref.	Ref.	Ref.
	Hearing impairment prior to meningitis	-2.58 (0.331)	0.11 (0.961)	-1.02 (0.708)	-1.28 (0.551)
	Onset of hearing impairment since meningitis	-5.34 (0.030)*	-1.57 (0.463)	0.507 (0.838)	-3.60 (0.078)

^1^for the environment domain: p-value estimation taking into account the heteroscedasticity of the model residuals;

^2^executive, senior executive, teacher, liberal profession;

^3^employee, worker

*p<0.05; p<0.01; /: the variable was associated with QOL with a p-value >0.20 in bivariate analyses and then not included in multivariate models

Only the final linear regression models including all groups of variables have been presented, the intermediate R^2^ represent the hierarchical contribution of each group.

#### Physical health

Multiple hierarchical linear regression analyses revealed that having a cancer and/or a chronic disease (B = -4.82, p = 0.029), having an unfavourable disability outcome (GOS) (B = -30.35, p<0.001) and onset of hearing impairment since meningitis (B = -5.34, p = 0.030) were independently associated with lower physical QOL (Table 3). It is worth noting that incident headache or worsening headache intensity at 12 months was associated with impaired physical QOL at a 10% p-value threshold (B = -4.51, p = 0.069). Explanatory power (R^2^) of this model was 0.55 (*p* < 0.001) (Table 3).

#### Psychological health

Being a woman (B = -4.36, p = 0.015), unfavourable disability outcome (GOS) (B = -15.31, p<0.001) at 12 months, incident headache or worsening headache intensity after meningitis onset (B = -5.62, p = 0.010) were independently associated with lower mental QOL (Table 3). Explanatory power (R^2^) of this model was 0.29 (*p* < 0.001) (Table 3).

#### Social relationships QOL

Being unemployed at meningitis onset (B = -12.60, p = 0.008), seizures before or during hospitalization (B = -5.94, p = 0.046) and unfavourable disability outcome at 12 months (GOS) (B = -9.62, p<0.001) were significantly associated with lower *social relationships* QOL independently from the other factors (Table 3). Explanatory power (R^2^) of this model was 0.20 (*p* < 0.001) (Table 3).

#### Environmental QOL

After p-value estimation adjustment due to heteroscedasticity of the model residuals, unfavourable disability outcome (GOS) at 12 months (B = -11.08, p<0.001) was independently associated with lower *environmental* QOL (Table 3). It is to be noted that being unemployed (B = -9.27, p = 0.054) and onset of hearing impairment since meningitis (B = -3.60, p = 0.078) were associated with lower social relationships QOL at a 10% p-value threshold. Explanatory power (R^2^) of this model was 0.25 (*p* < 0.001) (Table 3).

Adding variables related to CABM impairments in the four QOL’s models, significantly increase the explanatory power of multivariate models (for physical health R^2^ change = 0.42, p<0.001, for psychological health R^2^ change = 0.23, p<0.001, for social relationships R^2^ change = 0.06, p<0.001 and for environmental domain R^2^ change was 0.15, p<0.001) (Table 3).

## Discussion

In the present study, we showed that sequelae of meningitis remain common at 12 months in survivors, with more than 1 in 2 patients (53.9%) reporting incident/worsening headache and/or incident hearing impairment and/or unfavourable disability outcome. QOL scores for the physical health domain were far below the norm for the French population, with almost one in two patients (45.9%) having a quality of life score less than or equal to the 25th percentile of the general population score. Scores in other domains were comparable to those in general population [22, 23].

Few patients’ characteristics at the time of meningitis onset were associated with QOL at 12 months. However, it should be noted that being a woman was associated with lower physical and mental health QOL and being unemployed at meningitis onset was a predictor of lower QOL related to social relationships and environment. The impact of female gender on impaired QOL has already been shown in the literature [22, 24] as well as the negative impact of low-income on social functioning and satisfaction with living environment [24–26]. Regarding patients’ medical background, having a cancer and/or a chronic disease was an independent predictor of lower physical health QOL consistent with literature [22, 27]. Concerning CABM clinical course, in our study, seizures were associated with lower social relationships at 12 months post-CABM. The unpredictable nature of seizures that patients with CABM may have had experienced during acute CABM episode can be experienced as a traumatic event. This phenomenon is largely documented in epilepsy, while epileptic patients reported a notable prevalence of social anxiety (unpredictability occurrence of seizures in public) [28].

Some patients’ characteristics at meningitis onset are important to consider as they affect mid-term QOL. However, these factors explain only a small part of the QOL’s variance, and thus, they are not sufficient predictors of altered mid-term QOL. Then, systematic clinical follow-up of all patients should be carried out in order to anticipate and prevent the repercussions of meningitis on daily life.

The month-12 CABM disability outcome score (GOS) was the major determinant of the four QOL’s domains. Almost one third of patients had an unfavourable disability outcome in our study at 12 months, which was however lower than rates reported by other studies [1, 14, 29, 30]. These differences could be explained by different time intervals in these studies between the meningitis onset and the time of sequelae assessment, which most often occurs at discharge [1, 14, 29] or shortly after hospitalization [30]. In our study, we assessed disability outcomes at one year, leaving more time and opportunity for the patients to recover. This was corroborated by another study based on COMBAT patients, which found that modified Rankin score (mRs) improved between discharge and 12-month follow-up in most of CABM patients [7]. Most of the studies in literature investigated factors associated with disability outcome (GOS scores) [1, 7, 15, 29, 30]. Our study was a step beyond, by showing the determining impact of disability outcome on mid-term QOL.

Our study has shown an interesting association between headache (incident or worsening since meningitis) and CABM patients’ psychological health (p = 0.010) and physical health (p<0.10). We suppose that meningitis-related headache could be caused by cerebrospinal fluid (CSF) dynamics disorders. Actually, a prospective observational study found that CSF infection (CSF protein >2.5 g/l) (p<0.001) was significantly associated with the severity of headache in tuberculous meningitis [31]. We have previously identified that increased protein level was associated with unfavourable in-hospital outcome in another study from the COMBAT cohort [7]. Most of the studies focused on headache as a CABM revealing symptom and few investigated headache as a sequelae of CABM [12, 32]. These findings suggest the importance of considering medical management (detection, care and treatment) of headache after CABM to potentially maintain and prevent difficulties in physical and psychological functioning.

The prevalence rate of hearing impairment in our study of 28% is comparable to that found in other studies in the literature [6, 33, 34]. Despite the high prevalence of this impairment, we found very few data on how to manage this issue in adults with CABM. In our study, incident hearing impairment since meningitis was associated with impaired physical QOL (incident hearing impairment) (p = 0.030) and with environmental QOL (p<0.10). These findings underline that the sudden onset of hearing disorders may have a negative impact on QOL as people have not had time to adapt to it on a daily basis. These impairments may reduce access to information, to leisure activities, and to social relationships in daily-life that may cause loneliness and social isolation [35]. Consistent with our results, it was found in literature that hearing impairment, in general population, has an impact on QOL [36, 37]. Hearing impairment should be systematically detected in clinical practice as it could have impact on psychosocial functioning. Moreover, psychosocial impact of hearing impairment after CABM should be more widely investigated to apprehend the perceived consequences in patients’ daily lives and thus consider appropriate support and solutions.

In addition to our study, concerning other impairments due to meningitis, cognitive impairment was reported as impacting CABM patients QOL in a Dutch study [16]. Patients at 6 to 24 months from CABM showed lower mental health QOL, physical QOL and vitality compared to controls. Neuropsychological evaluations should be performed as early as possible after discharge from hospital as it may have consequences on psychosocial functioning.

### Strengths and limitations

We identified several limitations in our study. First, we collected data related to QOL only at 12 months. Then we did not have data regarding QOL at baseline, preventing us from making an intra-subject comparison between baseline and 12 months. It might also be interesting to have a longer-term follow-up of CABM patients to have more information on the support to be recommended. Second, since the findings were based on self-report measures collected by phone, it may have introduced a response bias, social desirability bias, even though questionnaires were anonymous and standardized. Moreover, questionnaires could be answered by proxies or by general practitioners that may have introduced a measurement bias, but fortunately this only concerns very few patients. Regarding our QOL models, except for physical health, variables included in models explain a relatively small proportion of the variance in QOL. The integration of psychosocial factors would enrich our models beyond the clinical characteristics of individuals.

## Conclusion

Adults CABM survivors included in our study presented CABM impairments, especially unfavourable disability outcome, incident hearing impairment and incident headache, that influence domains of QOL. Results from this observational study highlight psychological and psychosocial impact of CABM impairments, and the importance to consider subjective patients’ perception of CABM that may have an impact on QOL. Integrating the comprehension of psychosocial factors may help to better understand personal experience of CABM and improve patients’ health status. An early systematic detection and management of CABM impairments in all patients should be performed in clinical practice as early as possible and at various times after meningitis. A systematic assessment of QOL should be carried out at different post-meningitis times to identify levers to promote the psychological and psychosocial functioning of CABM people.

## Supporting information

S1 Table Bivariate analyses between independent variables and QOL’s domains. (DOCX)
