# Supplementary material for: Mid-term health-related quality of life in community-acquired bacterial meningitis survivors; the COMBAT study
Source: PLoS One. 2023 Mar 23;18(3):e0281544. doi: 10.1371/journal.pone.0281544 (PMC10035867; doi:10.1371/journal.pone.0281544)
Supplement: S1 Table — (DOCX) [file pone.0281544.s001.docx]

**Supporting information:**

**S1 Table. Bivariate analysis between independent variables and QOL’s domains**

| **Variables** | **Physical health QOL** | **Mental health QOL** | **Environmental QOL** | **Social relationships QOL** |
| --- | --- | --- | --- | --- |
| **Background characteristics** | | | | |
| Sex (Female) | 0.025^a^ | 0.039^a^ | 0.321^a^ | 0.593^a^ |
| Age | <0.001^b^ | 0.840 ^b^ | 0.755 ^b^ | 0.058 ^b^ |
| Social professional categories | <0.001^c^ | 0.174^c^ | 0.003^c^ | <0.001^c^ |
| **Health status** | | | | |
| Chronic diseases / Cancer (<5 years) | 0.003^a^ | 0.475^a^ | 0.394^a^ | 0.019^a^ |
| Alcohol abuse | 0.060^a^ | 0.647 ^a^ | 0.107^a^ | 0.007^a^ |
| Active smoking | 0.120 ^a^ | 0.078 ^a^ | 0.104^a^ | 0.970^a^ |
| Undernutrition | 0.642 ^a^ | 0.340 ^a^ | 0.610^a^ | 0.749^a^ |
| Recurrent meningitis | 0.942 ^a^ | 0.870 ^a^ | 0.582^a^ | 0.792^a^ |
| **CABM Clinical course** | | | | |
| Causative microorganisms | 0.031^c^ | 0.721^c^ | 0.767^c^ | 0.209^c^ |
| Corticoids | 0.940 ^a^ | 0.729 ^a^ | 0.761 ^a^ | 0.509 ^a^ |
| Admission in ICU | 0.864 ^a^ | 0.646 ^a^ | 0.060^a^ | 0.198^a^ |
| Seizures | 0.354 ^a^ | 0.185 ^a^ | 0.384^a^ | 0.009^a^ |
| Coma | 0.988 ^a^ | 0.078 ^a^ | 0.821^a^ | 0.781^a^ |
| **12-month follow-up CABM impairments** | | | | |
| GOS | <0.001 ^a^ | <0.001 ^a^ | <0.001 ^a^ | <0.001 ^a^ |
| Headache | <0.001 ^a^ | <0.001 ^a^ | 0.010 ^a^ | 0.107 ^a^ |
| Hearing impairment | <0.001 ^c^ | 0.038 ^c^ | 0.003^c^ | 0.376^c^ |

^a^Mann-Whitney; ^b^Pearson correlation; ^c^ANOVA
